# Deep-learning approach to stratified reconstructions of tissue absorption and scattering in time-domain spatial frequency domain imaging

**DOI:** 10.1117/1.JBO.29.3.036002

**Published:** 2024-03-12

**Authors:** Yaru Zhang, Wenxing Bai, Yihan Dong, Mai Dan, Dongyuan Liu, Feng Gao

**Affiliations:** a Tianjin University, College of Precision Instrument and Optoelectronics Engineering, Tianjin, China; b Tianjin Key Laboratory of Biomedical Detecting Techniques and Instruments, Tianjin, China

**Keywords:** time-domain spatial frequency domain imaging, deep learning, optical properties reconstruction

## Abstract

**Significance:**

The conventional optical properties (OPs) reconstruction in spatial frequency domain (SFD) imaging, like the lookup table (LUT) method, causes OPs aliasing and yields only average OPs without depth resolution. Integrating SFD imaging with time-resolved (TR) measurements enhances space-TR information, enabling improved reconstruction of absorption ($\mu_{a}$) and reduced scattering ($\mu_{s}^{\prime }$) coefficients at various depths.

**Aim:**

To achieve the stratified reconstruction of OPs and the separation between $\mu_{a}$ and $\mu_{s}^{\prime }$, using deep learning workflow based on the temporal and spatial information provided by time-domain SFD imaging technique, while enhancing the reconstruction accuracy.

**Approach:**

Two data processing methods are employed for the OPs reconstruction with TR-SFD imaging, one is full TR data, and the other is the featured data extracted from the full TR data (E, continuous-wave component, ⟨t⟩, mean time of flight). We compared their performance using a series of simulation and phantom validations.

**Results:**

Compared to the LUT approach, utilizing full TR, E and ⟨t⟩ datasets yield high-resolution OPs reconstruction results. Among the three datasets employed, full TR demonstrates the optimal accuracy.

**Conclusions:**

Utilizing the data obtained from SFD and TR measurement techniques allows for achieving high-resolution separation reconstruction of $\mu_{a}$ and $\mu_{s}^{\prime }$ at different depths within 5 mm.

## 1 Introduction

Spatial frequency domain (SFD) imaging technique is a large field-of-view optical imaging modality that employs a spatial light modulator to project structured patterns onto tissues for reconstructing the optical properties (OPs), including the absorption coefficient ($\mu_{a}$) and reduced scattering coefficient ($\mu_{s}^{\prime }$).^1^ In the conventional SFD imaging, tissues are simplified to the homogeneous single-layer structures.^2^^–^^8^ However, the challenge of reconstruction of $\mu_{a}$ and $\mu_{s}^{\prime }$ remains in the tissue optical imaging. Aliasing of depth information continues to be the primary cause of blurring in two-dimensional SFD imaging results. The issues of low depth resolution and accuracy in OPs reconstruction require immediate attention and resolution.

Some studies have developed forward transport models for multiple layers tissues based on the diffusion equation or Monte–Carlo (MC) simulation and have used a lookup table (LUT) method to reconstruct the OPs.^9^^–^^13^ The accuracy of the reconstructed multi-layer OPs, however, is limited by the sampling density in the precomputed dataset. More densely sampled data sets increase memory usage and slow computation. The diffuse optical tomography (DOT) technique allows for obtaining arbitrary OPs in space through the light propagation model and perturbation theory.^14^ But challenges still persist in the image reconstruction of DOT, mainly on the improvements of the spatial resolution and separation between the $\mu_{a}$ and $\mu_{s}^{\prime }$.^15^^,^^16^ SFD imaging with high spatial resolution in combination with the DOT reconstruction based on the diffusion model, has been shown to provide promising depth-resolved information of the turbid media.^17^ But the penetration depth of the light into tissues is limited at high spatial frequencies and the diffusion model may not be suitable in the near-field region, SFD-DOT suffers from inversion discomfort and a problem arises where the reconstructed region of interest is shifted towards the surface.^17^^–^^19^ The latest research reconstructed the three-dimensional (3-D) OPs by fusing micro-CT structural prior information and DOT for complex silicon and *in-vivo* imaging.^20^^,^^21^ Providing of the micro-CT structural prior information can effectively reduce the ill-posedness and non-linearity of the inverse problem in the DOT reconstruction. However, obtaining the structural prior information for OPs reconstruction is challenging in most cases.

Time-resolved (TR) imaging can enhance depth resolution and facilitate accurate reconstruction quantification. In TR measurements, the temporal profiles, which represent the temporal distributions of reemitted photons and contain refined information at varying depths, are measured. These profiles can provide more comprehensive information and help distinguish optical responses from targets at different depths.^22^ SFD imaging combined with TR measurement can provide complete and reliable temporal and spatial information for the reconstruction of stratified OPs. This information is expected to achieve high spatial resolution and improved depth resolution in tissue OPs reconstruction. In recent years, the deep learning (DL) method has been employed in SFD imaging, which is a model-independent reconstruction method that uses end-to-end neural network models to reconstruct OPs from diffuse reflectance images.^6^^,^^23^ Its unique high nonlinear fitting ability and strong adaptability enable strong performance in complex tasks.^21^ However, the limited measurement information provided by SFD imaging poses a challenge for improving reconstruction depth resolution. By leveraging sufficient information from TR-SFD measurement, DL can achieve high-resolution stratified reconstruction of OPs, effectively mitigating the ill-posedness inherent in DOT inverse problem and solving the problem of reconstructed region of interest being moved to the surface in SFD imaging reconstruction of OPs. Compared to other methods for reconstructing OPs mentioned above, DL can also achieve rapid reconstruction. In addition, simultaneous reconstruction of $\mu_{a}$ and $\mu_{s}^{\prime }$ can solve the aliasing problem and enable hierarchical reconstruction with different OPs.

In this work, we use a DL network architecture based on U-Net for reconstructing OPs.^24^ The network implements end-to-end stratified reconstruction of OPs and output 10 images representing $\mu_{a}$ and $\mu_{s}^{\prime }$ for five layers. The tissue within a depth of 5 mm is divided into five layers, with each layer representing a depth of 1 mm. We will process the TR data into two types of data. One type is the full TR data, while the other type is the featured data extracted from TR data. This featured data includes two different features: E representing the continuous-wave component, ⟨t⟩ representing the mean time of flight. The final obtained input dataset is diffuse reflectance images. LUT is the most widely available methods for SFD imaging to reconstruct OPs images. This study establishes the correlation between organizational OPs and diffuse reflectance data based on the MC–Hankel transform, forming a database.^1^ The value of $\mu_{a}$ ranges from 0.001 to $0.5\;{\mathrm{mm}}^{-1}$ with an interval of $0.001\;{\mathrm{mm}}^{-1}$, and for $\mu_{s}^{\prime }$, it ranges from 0.2 to $2.4\;{\mathrm{mm}}^{-1}$ with an interval of $0.01\;{\mathrm{mm}}^{-1}$. We also present the results of LUT and compared their performance through a series of simulations and phantom validations. Among all datasets, the full TR data performed the best and accurately distinguished OPs at different depth. In the anti-cross talk experiment, both ⟨t⟩ and full TR data effectively overcame cross talk interference. The phantom results demonstrate that all datasets can achieve varying levels of contrast OPs reconstruction, enabling stratified reconstruction of OPs. And all these results, have better spatial resolution and accuracy than those obtained by the LUT method.

## 2 Materials and Methods

### 2.1 Database Generation and Processing

A major challenge in DL is to create robust data generation workflows that can be applied to various architectures and imaging scenarios. MC is considered the gold standard for accurately modeling the propagation of light in tissues.^25^ Therefore, we utilized the GPU-based MC eXtreme program to generate TR-SFD data.^26^ In the program settings, $10^{9}\text{ photons}$ were emitted from a spatially modulated light source with varying spatial frequencies ($0.1\;{\mathrm{mm}}^{-1}$, $0.05\;{\mathrm{mm}}^{-1}$, and $0.025\;{\mathrm{mm}}^{-1}$), a total detection time of 1 ns. The simulated cuboids had dimensions of 60  mm×60  mm×30  mm and contained cylinders with radii ranging from 2 to 7 mm or shapes from the EMNIST dataset.^27^ The inclusions are randomly distributed at varying depths beneath the illuminated surface, with illumination and detection areas measuring $60\times 60\;{\mathrm{mm}}^{2}$ and $50\times 50\;{\mathrm{mm}}^{2}$, respectively. Referring to previous studies on the determination of the OPs value for biological tissues in visible light and near-infrared light,^28^^–^^33^ the background OPs of cuboid range from 0.001 to $0.5\;{\mathrm{mm}}^{-1}$ with an interval of $0.005\;{\mathrm{mm}}^{-1}$ for $\mu_{a}$, and from 0.5 to $2.2\;{\mathrm{mm}}^{-1}$ with an interval of $0.05\;{\mathrm{mm}}^{-1}$ for $\mu_{s}^{\prime }$. The $\mu_{a}$ values of inclusions fall within the same range as the background, while $\mu_{s}^{\prime }$ between 0.7 and $2\;{\mathrm{mm}}^{-1}$ with an interval of $0.05\;{\mathrm{mm}}^{-1}$. These OPs will be selected randomly for simulation. The diffuse reflectance is obtained through SFD imaging, which uses three-phase demodulation method to acquire the planar (DC) ($0\;{\mathrm{mm}}^{-1}$) and spatially modulated (AC) ($0.05\;{\mathrm{mm}}^{-1}$) component at different temporal gates.^1^

The TR measurement yields a temporal point spread function (TPSF) curve. To assess the consistency between the simulated and experimentally measured curves, homogeneous tissue phantoms with the same OPs will be used in the simulation and experimental measurements. The instrument response function (IRF) is experimentally collected and will be convolved with the curve obtained from the simulation.^34^ The simulated and experimentally measured tissue phantom parameters are as follows: the $\mu_{a}$ is $0.01\;{\mathrm{mm}}^{-1}$, the $\mu_{s}^{\prime }$ is $1\;{\mathrm{mm}}^{-1}$, the time resolution of the simulation experiment will be set to 12.2 ps according to the experimental time resolution. Both simulations and experiments were illuminated with planar light.

**Fig. 1:**
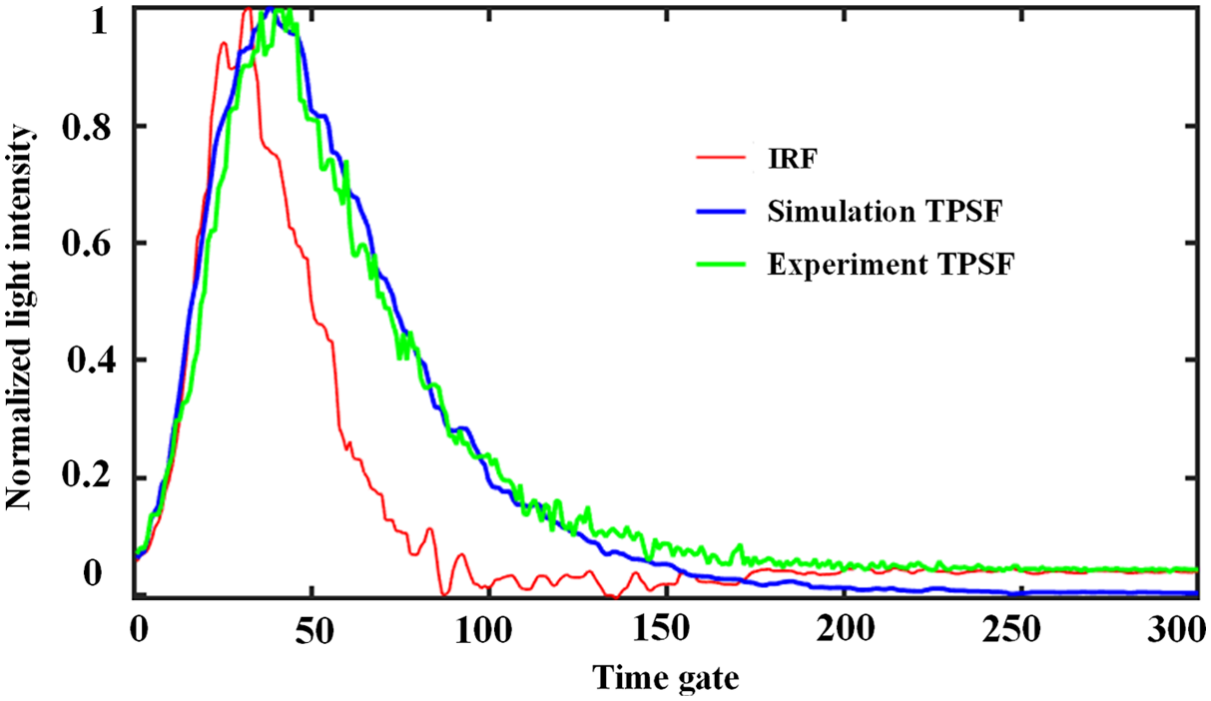
Comparison between experimental TPSF curve and simulated TPSF curve.

The results presented in Fig. 1 include three normalized intensity curves, the experiment TPSF curve, IRF curve, and the curve (shown in Fig. 1, “Simulation TPSF”) obtained by convolving the MC-simulated data with the IRF. The experiment TPSF curve and IRF are derived from the results of the system 780 nm measurements. In Fig. 1, all three curves are truncated to 300 temporal gates for comparison purposes. Furthermore, a close match and similar trends were observed between the convolved curve and the experimental curve, indicating the consistency between the post-processed TPSF curve from the simulated data and the measured curve.

In this study, we first obtain the featured data (⟨t⟩ and E) of the TPSF curve. E is given by Eq. (1) and ⟨t⟩ is given by Eq. (2)^35^

$$E=\int_{0}^{\infty }\Gamma dt,$$ (1)

$$\langle t\rangle =\frac{\int_{0}^{\infty }t\cdot \Gamma dt}{E},$$ (2)

where Γ represents a TPSF curve shape as “Experiment TPSF,” as shown in Fig. 1, and t represents time. Suppose the total number of temporal gates in one measurement is d. The width of each temporal gate is represented by g, and $y_{k}$ represents the photon intensity at the gate of k. Equations (1) and (2) are discretized to obtain Eqs. (3) and (4), respectively

$$E=\sum_{1}^{d-1}(y_{k}+y_{k+1})\cdot \frac{g}{2},$$ (3)

$$\langle t\rangle =\frac{\Gamma^{\left(1\right)}}{\Gamma^{\left(0\right)}}=\frac{\overset{d-1}{\underset{1}{\sum }}(y_{k}+y_{k+1})\cdot \frac{g}{2}\cdot (k\cdot g-\frac{g}{2})}{\overset{d-1}{\underset{1}{\sum }}(y_{k}+y_{k+1})\cdot \frac{g}{2}}.$$ (4)

Additionally, the utilization of full TR dataset enables enhanced spatial resolution and improved quantitative accuracy in OPs reconstruction.^16^

DC and AC images are obtained by processing the TPSF curves to get full TR data and featured data and subsequently demodulating them. Eventually, the diffuse reflectance images will be obtained.^1^ For ease of subsequent description, full TR, E, and ⟨t⟩ data will be used to represent the different datasets. To ensure a balanced influence on the loss function, $\mu_{s}^{\prime }$ and $\mu_{a}$ values are distributed between 0 and 1.^36^

In summary, three types of data will be input into the network. These three datasets are trained separately to explore the influence of different types of data on the reconstruction result of OPs.

### 2.2 U-Net

**Fig. 2:**
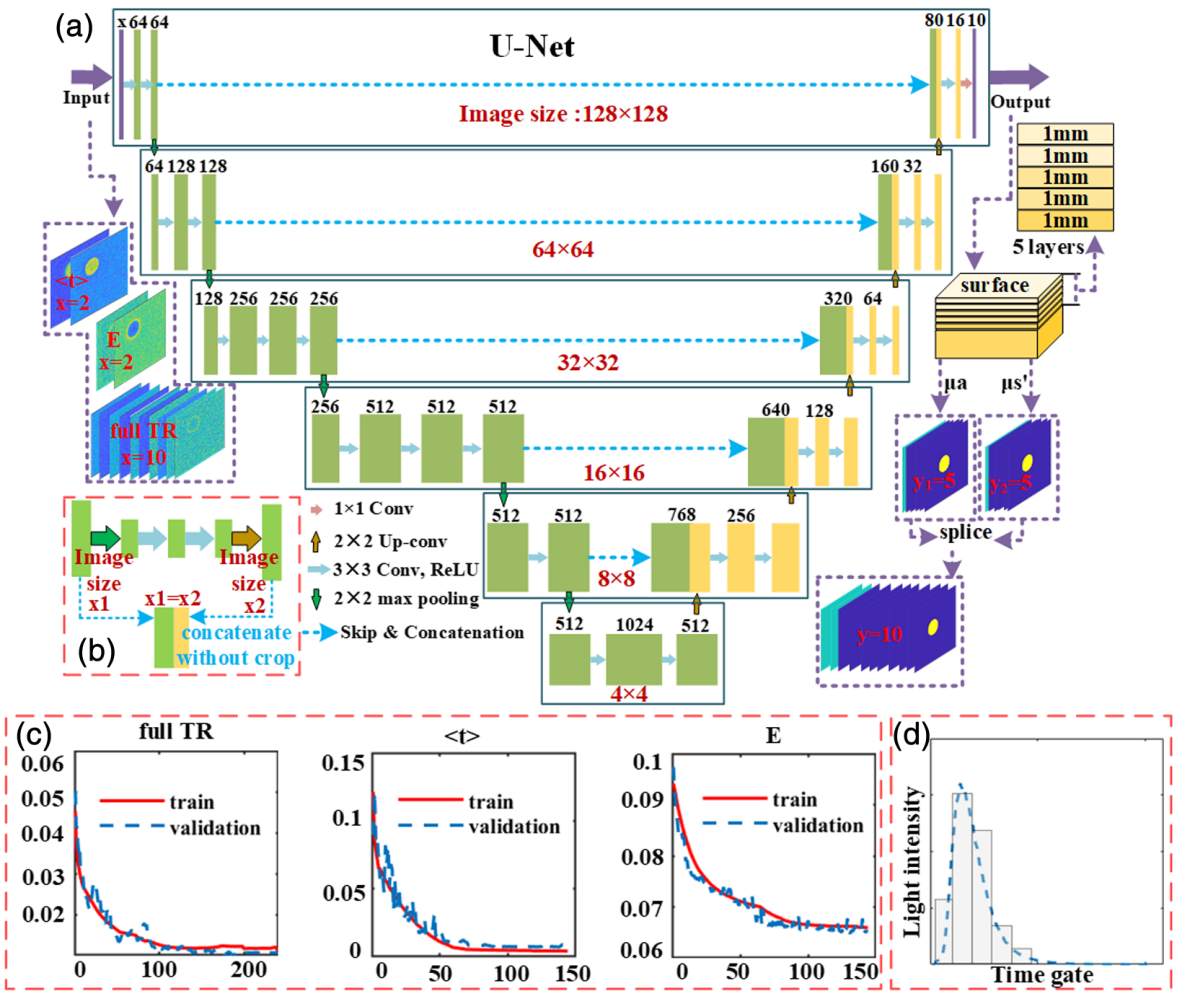
Network architecture: (a) the U-Net network structure used in this study and (b) the schematic of each skip connection. (c) Loss-Epoch curve, its horizontal axis represents every 5 Epochs by taking a loss value. (d) Temporal profile was divided into equally about five time slices.

A common application of convolutional networks is image classification, while in biomedical image reconstruction, the focus is on localization and quantification. In other words, each pixel in an image needs to be assigned a class tag. U-Net, a widely used convolutional neural network structure.^24^ It is particularly suitable for scenarios requiring consideration of local information and high-resolution details. In this study involving the OPs of different regions within images, the skip connections in U-Net assist the network in capturing local information more effectively. Its network structure can extract the spatial distribution features of images well and is very suitable for such image-to-image input and output training tasks. Therefore, the U-Net shown in Fig. 2(a) is employed in this study to extract depth information from both full TR data and featured datasets for achieving the task of stratified reconstruction of OPs.

The dataset consists of images with dimensions of 128×128  pixels. In one experiment, before the three-phase demodulation, the data were distributed on different time gates, and there were three phases respectively, and the data size was 128×128×3×d where d is the number of temporal gates. Then the TR data of each phase are processed, as shown in Fig. 2(d), all temporal gates are divided into five temporal segments, and the values of each segment are directly summed, then the data obtained is 128×128×3×5. Subsequently, a three-phase demodulation operation was performed for each time segment, and the demodulated data included DC and AC data. At this point, the data obtained is 128×128×2×5, which is 128×128×10. Therefore, the shape of the full TR dataset was 4676×128×128×10. As for E and ⟨t⟩, and it is when the data size is 128×128×3×d, using the Eq. (3) and (4) to obtain the featured data, the size becomes 128×128×3×1, and then the three-phase demodulation operation is carried out, the data obtained is 128×128×2×1, which is 128×128×2. Therefore, the input of featured data size is 4676×128×128×2. The above 2 and 10 correspond to the number of channels input by the network. The output includes OPs of five tissue layers within a depth of 5 mm. Each layer has a thickness of 1 mm and contains one $\mu_{s}^{\prime }$ and one $\mu_{a}$ image. The image resolution is 128×128, making the size of the output data 4676×128×128×10. 10 is corresponding to the network’s last layer 1×1 convolution of the size of the output channel.

The time dimension information is disassembled operation by operation to obtain detailed feature maps, and the maximum pooling operation eliminates redundant information from the convolution, allowing for the extraction of spatial information through the core flow of the network. We have avoided information loss caused by clipping upper feature maps during skip connections by processing input and output directly at the same picture resolution. The traditional skip join converts the data from the time dimension to the spatial dimension by clipping the under-sampled feature map to match the size of the deep feature map.^24^ However, in our U-Net network, we convert diffuse reflection OPs images data from the time dimension to the spatial dimension in skip connections without any changes in image size. That is, after each up-sampling, the image size is changed to the same size as the image before each pooling operation in the feature extraction operation, and Fig. 2(b) shows one of the skip connections.

The simulated and phantom datasets were divided into a ratio of 8:2. For training and testing purposes, the data were split in a ratio of 6:4 to improve network performance and prevent overfitting. To optimize the final saved network model while reducing training time, the training set was randomly shuffled and a maximum Epoch value was used. If the maximum Epoch value was reached without any further decrease in loss, the training would be stopped and the model saved. Keras with a Tensorflow backend was utilized for training the network, using an Adam optimizer with a learning rate of 0.001 and the batch size is 12. The loss function employed was mean squared error. As shown in Fig. 2(c), throughout the training process, the results obtained from training with these three datasets are convergent, and the trends of the validation set loss and training set loss curves are essentially consistent. At the same time, the similarity in loss values suggests that the model exhibits good robustness to different datasets. GPU acceleration techniques were utilized during the training process.

### 2.3 Imaging System

**Fig. 3:**
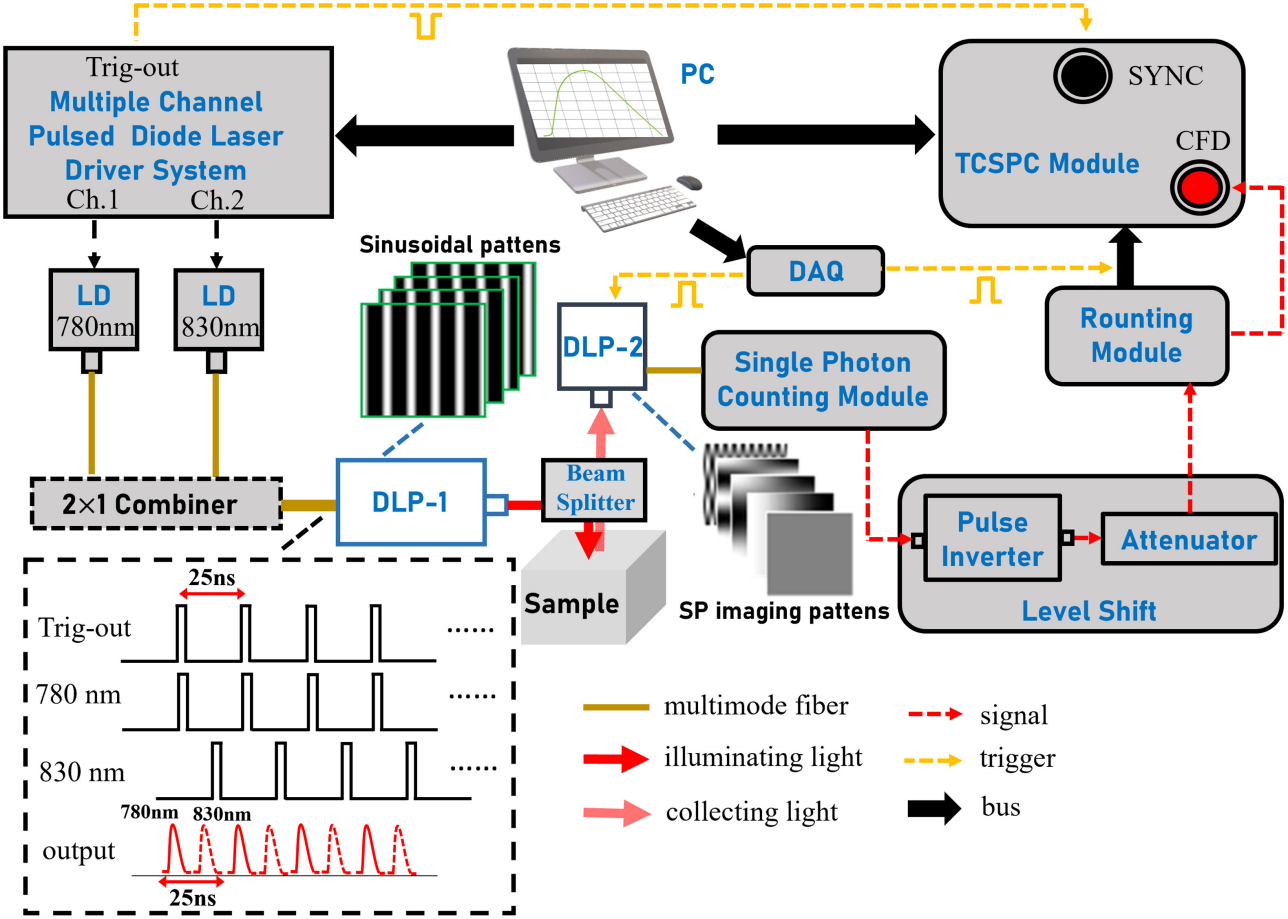
Schematic of TR-SFD imaging system.

The system schematic is shown in Fig. 3. Two digital micromirror devices (DMD) enabling structural light illumination for SFD imaging and single pixel (SP) compression encoding for diffuse reflection signal. DMD-1 and DMD-2 are respectively housed in DLP-1 and DLP-2. The light passes through a beam homogenizer and shaper before being transmitted onto the DMD by a DLP projector, and subsequently reflected by the projection lens.

The system utilizes a software-controlled multi-channel picosecond semiconductor laser driver (PDL 828 “Sepia II,” PicoQuant GmbH, Germany) to drive two laser heads (LDH-P-780, LDH-P-830, PicoQuant GmbH, Germany). The maximum operating frequency of the system is 80 MHz. In serial mode, the two lasers sequentially excite pulsed light with a repetition rate of 40 MHz. With a repetition period of 25 ns, laser pulses at wavelengths of 780 nm and 830 nm are generated in sequence. When excited simultaneously at a repetition frequency of 40 MHz, successful separation of multi-wavelength signals can be achieved. Two wavelengths are combined into one beam and directed to the spatial modulator (E4500 MKII, EKB Technologies, Israel) of DMD-1 for spatial encoding. The encoded light generates a sinusoidal stripe pattern with 256-level, which is projected onto the sample surface for measurement.

During acquisition, diffuse light from the object passes through a lens and undergoes spatial modulation by DMD-2. In this study, the DMD-2 is employed to generate a sequence of sampled observation matrices required for SP imaging. Considering that it is not feasible for DMD-2 to directly upload negative number codes, the two-dimensional observation matrix will be split into positive and negative parts in actual measurement.^17^ The SFD reflectance images was measured using single-pixel imaging based on two-dimensional discrete cosine transform (DCT), which spatially compressed the pixel-array images using the sampling patterns composed of the DCT kernel matrices and recovered the images by applying an inverse DCT to the DCT coefficients acquired from the SP detector.^37^ The DCT sampling template is partially shown in Fig. 3. In order to reduce the number of sampling patterns, the images are recovered by a selective acquisition of DCT coefficients concentrated in DC component and AC modulation frequency that fully utilizes the sparsity of sine harmonic in cosine base. Each DCT coefficient is obtained using the corresponding DCT kernel matrix. The number of pixels of the recovered image is 128×128. Tissue optical imaging has low-pass filtering characteristics, and the information of light scattered by photons entering the tissue is filtered to the low-frequency band, which contains less high-frequency information. Therefore, using the sampling of DCT at low frequency can obtain the measurement results beneficial to our study.

In this study, the integrated light signal is focused into a multimode fiber using achromatic twin lenses and detected by a fiber-coupled avalanche photodiode (SPCM-AQRH-13-FC, Excelitas Technologies, Canada) for single photon counting. The electrical pulse output of the detector is subsequently directed to the time-correlated single photon counting (TCSPC) module for precise TR data collection. Additionally, by combining the synchronization signal of the laser driver with the trigger signal of the spatial light modulator, signals of different wavelengths and acquisition masks can be separated accordingly. The combination of SFD and TCSPC imaging represents a powerful TR measurement technique that offers wide field-of-view, high sensitivity, and large dynamic range capabilities, thereby providing accurate photon transport information for generating high-quality tomographic images.

## 3 Results

To scientifically evaluate network performance in OPs reconstruction, three evaluation metrics will be selected: normalized mean absolute error (NMAE), average optical property (AOP) where a lower value indicates a smaller reconstruction error,^6^ and quantitativeness ratio (QR) where a value approaching 1 indicates smaller error. The simulation and phantom results from all test sets will be presented, followed by a comparison and analysis of the effects that these three data have on OPs reconstruction. In the following, NMAE1 and NMAE2 will be used to represent the NMAE of reconstructed $\mu_{a}$ and $\mu_{s}^{\prime }$. QR1 and QR2 are also the same. The QR is given by the following equation:

$$\mathrm{QR}=\max(\mu_{\mathrm{pre}})/\max(\mu_{\mathrm{tru}})\times 100\%,$$ (5)

where $\mu_{\mathrm{pre}}$ represent the reconstruction values of the $\mu_{a}$ or $\mu_{s}^{\prime }$, and $\mu_{\mathrm{tru}}$ is the true OPs value.

**Fig. 4:**
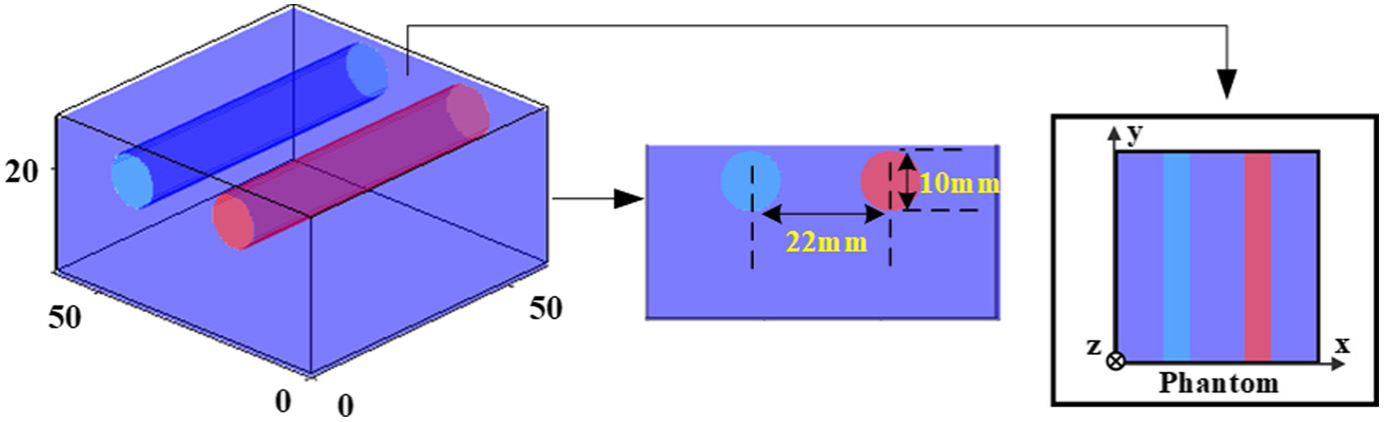
Sketch of the tissue phantom.

Figure 4 illustrates a 3-D distribution map, based on which simulated and phantom data for OPs reconstruction can be obtained. The diameter of both cylinders is 10 mm, the distance between their centers is 22 mm, and they are both located at the distance of 1 mm from the illumination and detection surface (the upper surface). The middle of the figure represents the side view, while the far right depicts the top view.

### 3.1 Simulations

In simulation results, using the 3-D map in Fig. 4, the spatial frequency is set to $0.05\;{\mathrm{mm}}^{-1}$, with OPs configured at a background $\mu_{a}$ value of $0.01\;{\mathrm{mm}}^{-1}$ and a $\mu_{s}^{\prime }$ value of $1\;{\mathrm{mm}}^{-1}$. Additionally, there are inclusions (the right cylinder and left cylinder) with $\mu_{a}$ values of $0.015\;{\mathrm{mm}}^{-1}$ and $0.02\;{\mathrm{mm}}^{-1}$, along with $\mu_{s}^{\prime }$ values of $1.5\;{\mathrm{mm}}^{-1}$ and $2\;{\mathrm{mm}}^{-1}$.

**Fig. 5:**
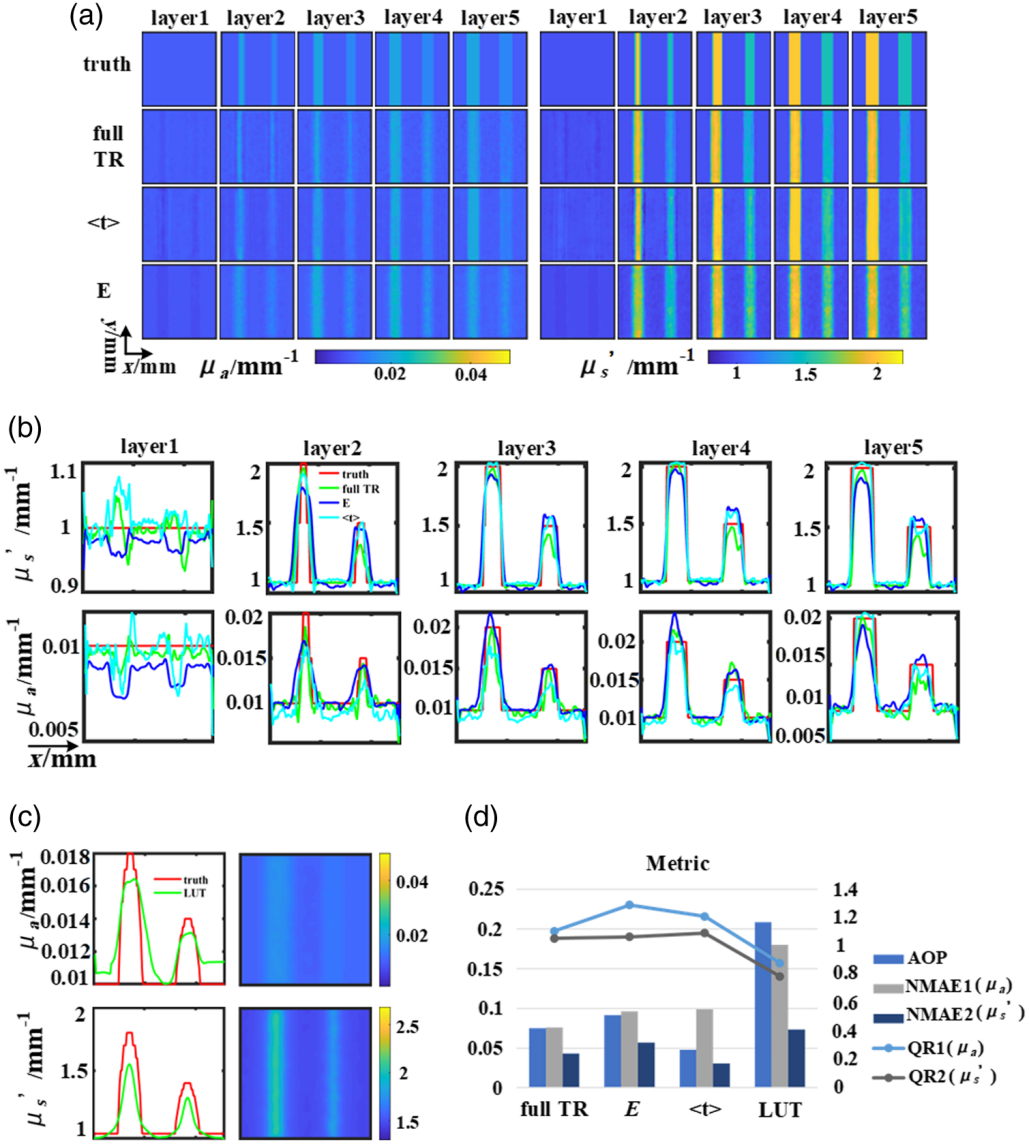
Results of the reconstruction of the two cylinders: (a) results of the U-Net. (b) Line-profiles of horizontal cross-sections. (c) Results of LUT. (d) The evaluation index of the reconstructed image, line graph corresponds to the right coordinate.

The reconstruction results of the simulated data in Fig. 5 validate the effectiveness of the network model in accurately reconstructing OPs within a 5 mm margin. However, when using featured data E, the width of the inclusions in each layer of $\mu_{a}$ is either too narrow or too wide (in layer 2), compared to their accurate size, and the reconstructed results were larger than the truth values. The size of the inclusions can be accurately identified using featured data ⟨t⟩. However, in layer 1, some values were inaccurately assigned to positions where the inclusions do not exist using E and ⟨t⟩. The reason mainly lies in the insufficiency of the information content in the featured data sets.^17^ Compared to the reconstruction result of the featured data ⟨t⟩ and E, the reconstruction result of the full TR data eliminates artifacts and improves the accuracy of reconstructing different layers. Specifically, for $\mu_{s}^{\prime }$, all three datasets can be reconstructed with high accuracy, in layer 2, the results using featured data ⟨t⟩ are more accurate in determining inclusion size. The further analysis of the profile diagram along the transverse section [Fig. 5(b)] reveals that in the reconstruction results of different layers, the full TR data yield the most favorable outcome. The largest error in reconstruction accuracy is found in E.

Reconstruction results obtained through LUT are presented in Fig. 5(c). The truth values represent the averaging of OPs values within 5 mm. The results demonstrate the efficacy of LUT for topological reconstruction of OPs, while the stratified reconstruction of OPs cannot be achieved. The above results were further verified based on the evaluation index in Fig. 5(d). Specifically, the training results of using three datasets show that the NMAE1, NMAE2, and AOP of E are the largest, and QR1 is the farthest from 1. The utilization of full TR yields the best resolution results. These results are expected because the dataset of full TR contains more information that can help distinguish OPs at different depths. However, since the processing of E ignores the advantage brought by TR, it performs the worst in terms of the reconstruction task among the three datasets. The processing method of ⟨t⟩ only utilizes partial information from TR, therefore, the reconstruction results are not as good as those using full TR. Compared with LUT, the reconstruction error of the three datasets is smaller and the resolution is higher.

**Fig. 6:**
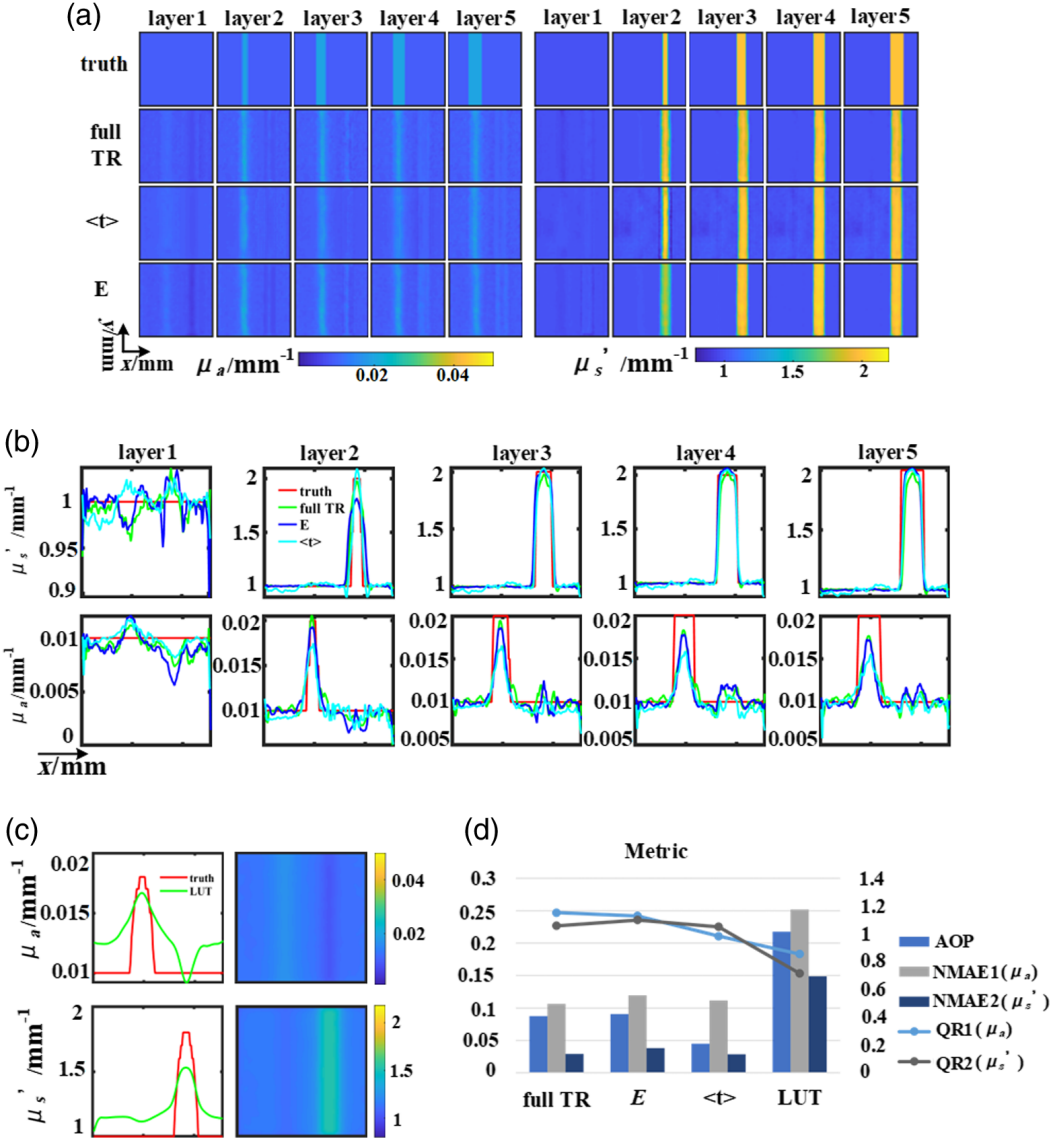
Verification of the reconstruction of anti-cross talk: (a) ground truth and results of the U-Net. (b) Line-profiles of horizontal cross-sections. (c) Results of LUT. (d) The evaluation index of the reconstructed result, line graph corresponds to the right coordinate.

The simulation experiments also validated the effectiveness of using three datasets in reducing cross talk influence on reconstructed $\mu_{a}$ and $\mu_{s}^{\prime }$. The distribution of inclusions and the OPs of background are the same as those in Fig. 4, the spatial frequency is set to $0.05\;{\mathrm{mm}}^{-1}$, with OPs set at a background $\mu_{a}$ value of $0.01\;{\mathrm{mm}}^{-1}$ and a $\mu_{s}^{\prime }$ value of $1\;{\mathrm{mm}}^{-1}$, along with inclusions (the right cylinder and left cylinder) having $\mu_{a}$ values of $0.01\;{\mathrm{mm}}^{-1}$ and $0.02\;{\mathrm{mm}}^{-1}$, and $\mu_{s}^{\prime }$ values of $2\;{\mathrm{mm}}^{-1}$ and $1\;{\mathrm{mm}}^{-1}$, respectively. The U-Net demonstrates remarkable efficacy in reducing impact of cross talk shown in Fig. 6(a) compared with LUT. Notably, the reconstruction results using the full TR data are almost unaffected by cross talk, while other featured data are influenced to varying degrees by cross talk [Fig. 6(a)].

As illustrated in Fig. 6(b), reconstructions of $\mu_{a}$ and using E exhibit pronounced cross talk. Specifically, the reconstruction of $\mu_{s}^{\prime }$ with E and full TR exhibits inaccuracies in determining the size of the inclusions. While the utilization of full TR effectively mitigates cross talk interference in layers 2 and 3, it remains challenging to eliminate cross talk in layers 4 and 5. Compared to using the results of full TR, utilizing the results of ⟨t⟩ can also alleviate the effects of cross talk to some extent. However, when reconstructing deeper layers (layers 4 and 5) of $\mu_{a}$, it is also difficult to eliminate the effects of cross talk completely.

The results of the LUT are shown in Fig. 6(c). Clearly, the LUT has a poorer ability to eliminate interference. Furthermore, in Fig. 6(d), the evaluation metrics reveal that employing featured data extracted from TR data leads to increased reconstruction errors (higher NMAE1 and NMAE2 values) compared to the use of full TR data. The LUT reconstruction results perform the worst.

### 3.2 Phantom Experiments

**Fig. 7:**
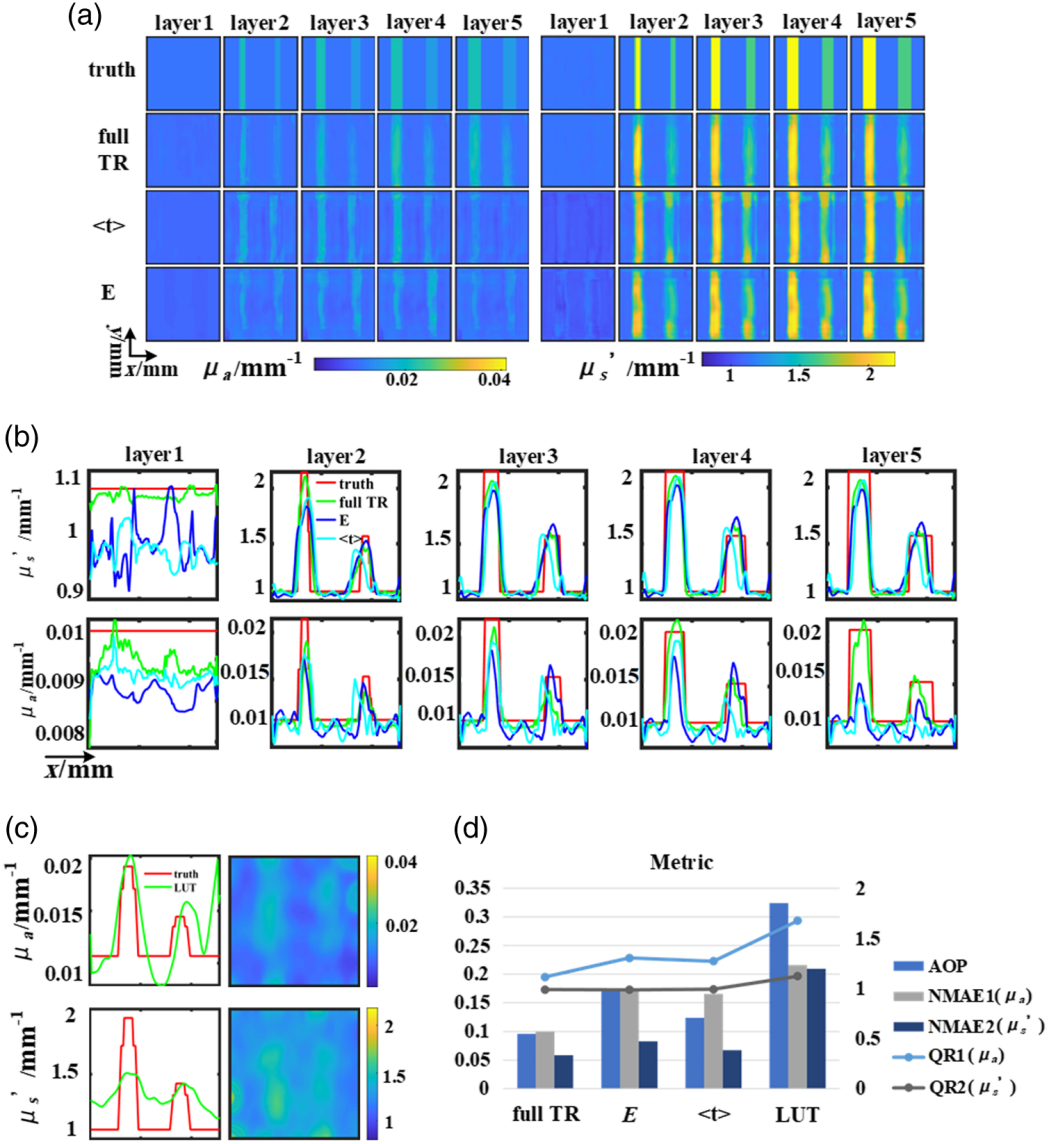
Verification of the phantom reconstruction: (a) ground truth and results of the U-Net. (b) Line-profiles of horizontal cross-sections. (c) Results of LUT. (d) The evaluation index of the reconstructed result, line graph corresponds to the right coordinate.

The presented results from the liquid phantom experiment, conducted at a measurement wavelength of 780 nm, and employing a spatial frequency of $0.05\;{\mathrm{mm}}^{-1}$, are depicted in Fig. 7. Tissue-like liquid phantoms were fabricated for validation, using India ink as absorber, titanium dioxide (TiO2) as scatterer. Notably, the background of the phantom is characterized by $\mu_{a}$ value of $0.01\;{\mathrm{mm}}^{-1}$ and $\mu_{s}^{\prime }$ value of $1\;{\mathrm{mm}}^{-1}$. The inclusions within the phantom exhibit differing $\mu_{a}$ values of $0.015\;{\mathrm{mm}}^{-1}$ and $0.02\;{\mathrm{mm}}^{-1}$, coupled with $\mu_{s}^{\prime }$ values of $1.5\;{\mathrm{mm}}^{-1}$ and $2\;{\mathrm{mm}}^{-1}$, for the right and left cylindrical inclusions respectively. The homogenous reference phantom parameters in the experiment are: $\mu_{a}$ value of $0.01\;{\mathrm{mm}}^{-1}$ and $\mu_{s}^{\prime }$ value of $1\;{\mathrm{mm}}^{-1}$. It is worth emphasizing that two horizontal cylinders, each with a diameter of 10 mm, were meticulously positioned at a distance of 1 mm from the surface of illumination, as delineated in Fig. 4. Subsequently, these cylinders underwent measurement via the TR-SFD imaging system shown in Sec. 2.3.

The reconstruction results of full TR data demonstrate its superior efficacy in distinguishing between OPs across various layers, as elegantly showcased in Fig. 7(b). This superiority primarily stems from the rich temporal information encapsulated within TR data, inherently corresponding to diverse depth distributions. Previous scholarly endeavors have validated the favorable outcomes attained through the utilization of full TR data, eclipsing the efficacy of featured data types.^16^ Moreover, in different contrast reconstruction results, DL manifests a remarkable capacity for discriminating between the $\mu_{s}^{\prime }$ in contrast to the $\mu_{a}$.

Consequently, compared with the reconstruction results of simulated data, the reconstruction results of phantom data have a higher incidence of artifacts. As illustrated in Figs. 5 and 7, through the analysis of the results, it was observed that the background of the reconstructed phantoms contains considerable amounts of artifacts, particularly evident in the outcomes derived from the LUT method. This phenomenon is also present in the results obtained using the featured dataset. However, when utilizing the full TR dataset, the occurrence of artifacts is almost negligible. It is also noted that there is a deviation between the true and reconstructed values, particularly in the layer 1 of reconstruction when utilizing featured data. The reason mainly lies in the insufficiency of the information content in the featured datasets.

**Fig. 8:**
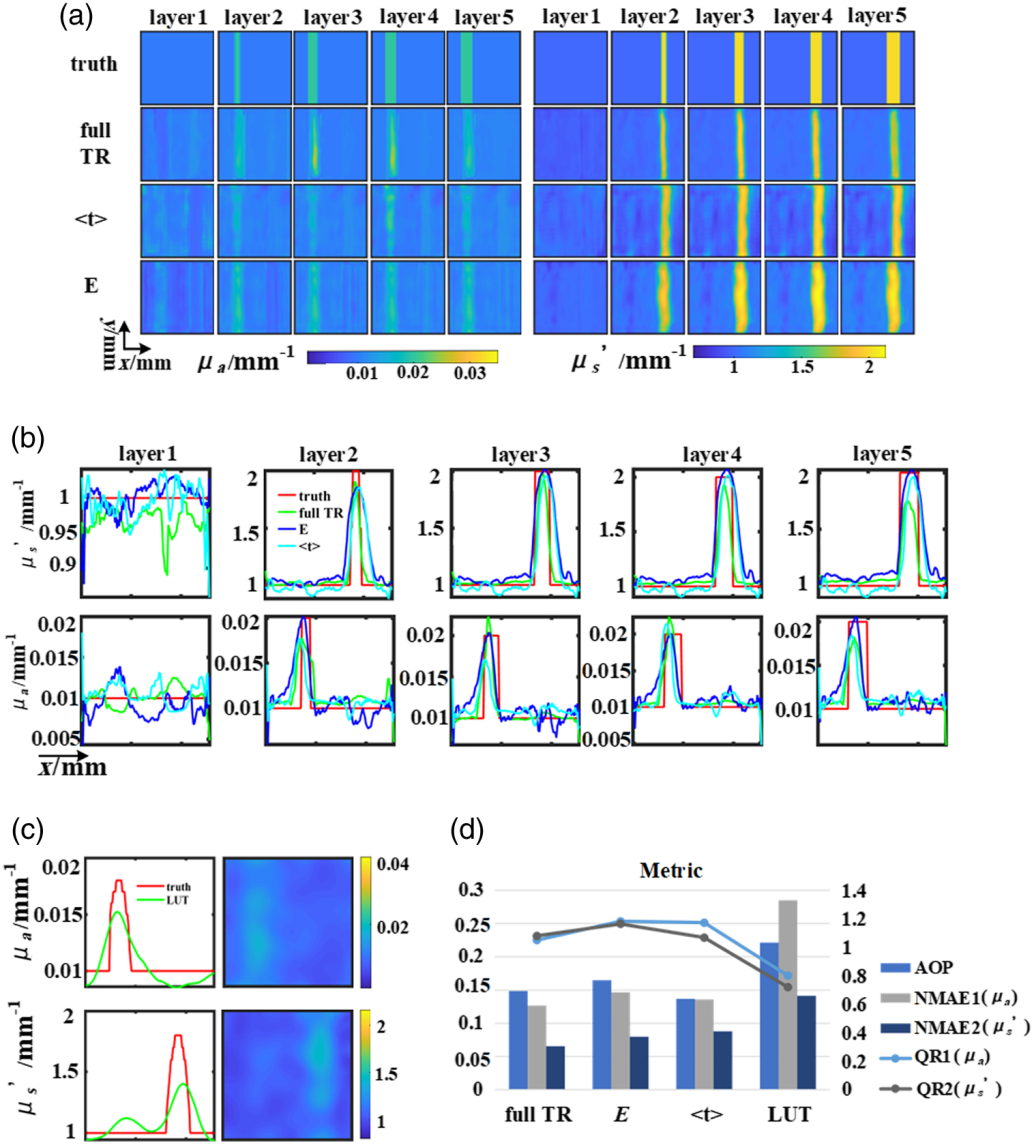
Verification of anti-cross talk in phantom reconstruction: (a) ground truth and results of the U-Net. (b) Line-profiles of horizontal cross-sections. (c) Results of LUT. (d) The evaluation index of the reconstructed result, line graph corresponds to the right coordinate.

We also measured another liquid phantom using the imaging system shown in Fig. 3. This phantom also utilized the structure shown in Fig. 4, the background of OPs set at a $\mu_{a}$ value of $0.01\;{\mathrm{mm}}^{-1}$ and a $\mu_{s}^{\prime }$ value of $1\;{\mathrm{mm}}^{-1}$, along with inclusions (the right cylinder and left cylinder) having $\mu_{a}$ values of $0.01\;{\mathrm{mm}}^{-1}$ and $0.02\;{\mathrm{mm}}^{-1}$, and $\mu_{s}^{\prime }$ values of $2\;{\mathrm{mm}}^{-1}$ and $1\;{\mathrm{mm}}^{-1}$ respectively. The reconstruction results are shown in Fig. 8 for a measurement wavelength of 780 nm and a spatial frequency of $0.05\;{\mathrm{mm}}^{-1}$. The reconstruction results show that, consistent with the performance of the simulation experiments, the reconstruction results of the model trained with full TR at different layers outperform the results of featured data. The errors of the reconstructed OPs become larger as the depth increases in Fig. 8(b), indicating that the model trained using full TR exhibits different sensitivities to OPs at different depths.

Comparison of the results of the two featured data reveals that the reconstruction results using E do not differ much numerically from layer to layer, whereas the results of ⟨t⟩ vary considerably with depth. The results of E show cross talk, whereas this phenomenon is almost absent in ⟨t⟩, which also shows that the use of ⟨t⟩ does ameliorate the effect of cross talk compared with using E. However, the overall results of ⟨t⟩ show greater inhomogeneity in the background locations compared to full TR, which may be caused by the different amount of information carried by the dataset itself. Compared with the reconstruction results of the simulation phantom, the reconstruction errors of the measurement phantom and the background are large. And in Fig. 8(c), the LUT results are also affected by cross talk, and the edge position of the variant is not clear enough. The evaluation metrics of the reconstruction results of different data types are also deteriorated accordingly in Fig. 8(d).

## 4 Discussions

The inherent limitations of the traditional methodology have been highlighted for OPs reconstruction, particularly in regard to its depth and precision. In stark contrast, DL stands as a model-independent methodology that is impervious to physical approximations, and excels in the attainment of high-resolution and high-precision OPs reconstructions characterized by robustness. The results of this study demonstrate that DL effectively utilizes the temporal and spatial information provided by the combination of SFD and TR, resulting in clearer and more realistic reconstructions compared to the method LUT. Comparative analysis of stratified OPs reconstruction was conducted employing two distinct datasets: full TR data and featured data. The results derived from full TR data revealed better depth resolution, attributed to its containing of a majority of photon transmission information. In particular, the results for the full TR data show better results for both the $\mu_{a}$ and $\mu_{s}^{\prime }$ reconstructions. While single featured data may exhibit relatively inferior quantization and depth resolution, it confers the advantage of substantially reducing input dimensionality for the DL. The traditional LUT method does not have the ability of stratified OPs reconstruction. Moreover, its reconstruction accuracy is also the lowest in this study.

**Fig. 9:**
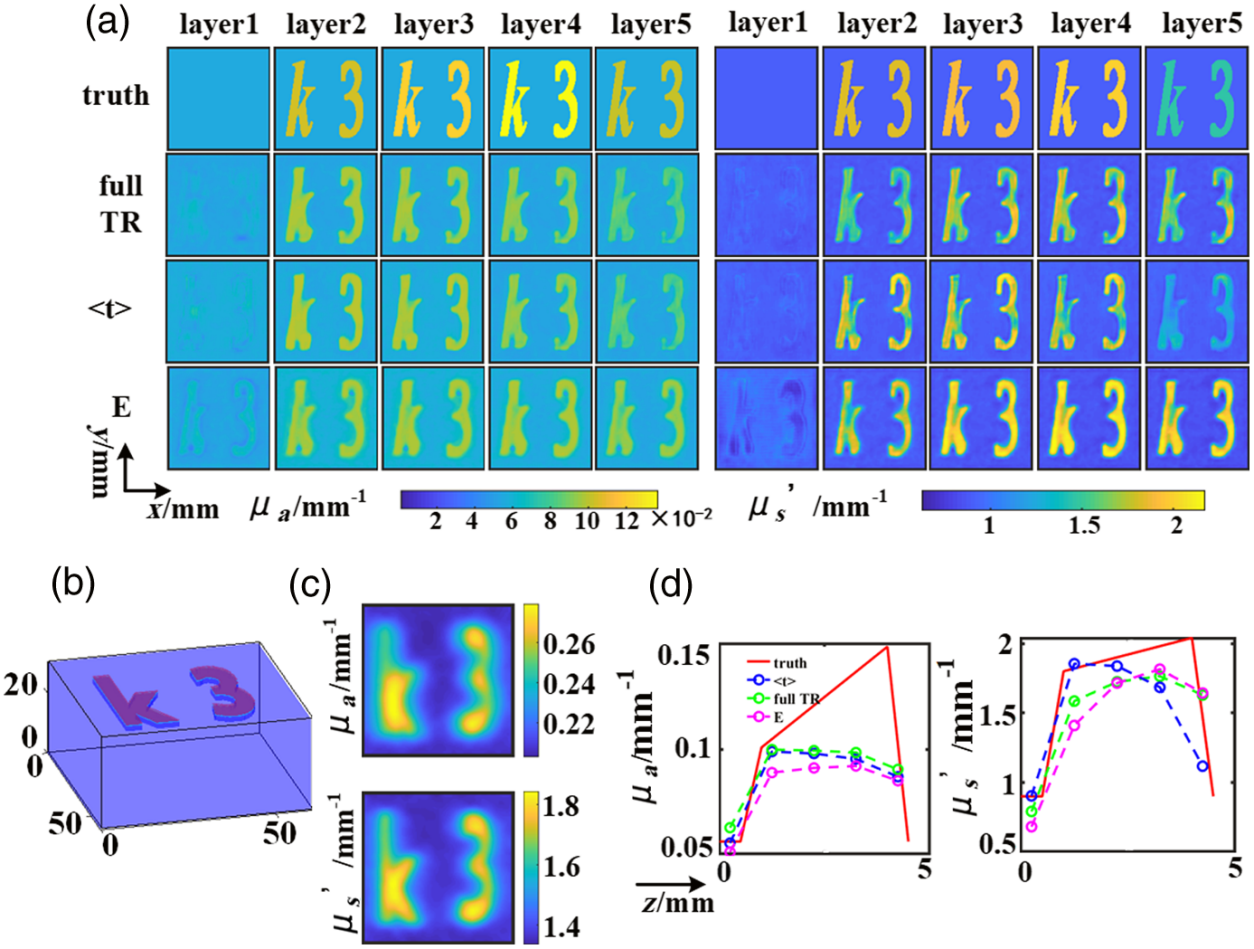
OPs reconstruction with increasing values: (a) results of the U-Net. (b) Simulation model sketch. (c) Results of LUT. (d) z line-profiles of vertical cross-sections at y=15  mm, x=35  mm.

The findings of this study encompass the reconstruction of OPs characterized by diverse contrasts and depths. Notably, the OPs of inclusions embedded within actual tissue are not constant across different depths. Therefore, datasets simulating the gradual variations in OPs at various depths were incorporated to emulate the OPs distribution within real tissue. These reconstruction results are vividly presented in Fig. 9, wherein two inclusions are positioned at a 1 mm depth from the illumination surface. In Fig. 9, the depth of 5 mm from the detection surface is subdivided into 10 discrete layers for the assignment of OPs. Subsequently, the OPs within each 1 mm layer are summed and averaged to obtain standard values. The background medium in this context is characterized by $\mu_{a}$ value of $0.056\;{\mathrm{mm}}^{-1}$ and $\mu_{s}^{\prime }$ value of $0.9\;{\mathrm{mm}}^{-1}$. The specific values for “k” and “3” are as follows: $\mu_{a}$: 0.1008, 0.1088, 0.1168, 0.1248, 0.1328, 0.1408, 0.1488, and $0.056\;{\mathrm{mm}}^{-1}$; and $\mu_{s}^{\prime }$ :1.8, 1.84, 1.88, 1.92, 1.96, 2, 2.04, and $0.9\;{\mathrm{mm}}^{-1}$ respectively, spanning a total depth of 4 mm and encompassing eight unique OPs for each depth level. The standard OPs values for “k” and “3” are: 0.1048, 0.1208, 0.1368, and $0.1024\;{\mathrm{mm}}^{-1}$ for $\mu_{a}$ and 1.82, 1.9, 1.98, $1.47\;{\mathrm{mm}}^{-1}$ for $\mu_{s}^{\prime }$. The network model showcased its robustness in reconstructing OPs with varying values at different depths (as depicted in Fig. 9). The results obtained by the LUT method are displayed in Fig. 9(c).

The OPs reconstruction results for vertical cross-sections at y=15  mm, x=35  mm are presented in Fig. 9(d). The red line represents the ground truth values, which were established by dividing different layers into ten segments with thickness of 5 mm, as shown in Fig. 9. Notably, the reconstruction results for $\mu_{s}^{\prime }$ exhibit closer concordance with the true values, whereas $\mu_{a}$ demonstrates increasing errors with depth. This discrepancy can be attributed to the contrast factor, as $\mu_{s}^{\prime }$ exhibits a larger contrast value compared to $\mu_{a}$. However, the model adeptly mitigates aliasing effects in depth-related OPs, and the variances in reconstruction values across different layers underscore the capability of the network in OPs reconstruction at varying depths, notwithstanding the relatively diminished accuracy in $\mu_{a}$ at deeper layers. In summation, among all the results, the reconstructions stemming from full TR data consistently exhibit the most exceptional performance.

The research has several limitations. When compared to simulation phantom results, the phantom results exhibit a higher number of reconstruction artifacts, except for cases involving full TR data. Furthermore, despite the simulation program encompassing experiments involving a diverse range of shapes, including cylinders, numbers, and letters, we were unable to obtain a large number of phantom data due to the time-consuming nature of equipment data acquisition. It has to be admitted that from the reconstruction results, our current model is flawed in that we are unable to completely accurately reconstruct the OPs of homogeneous layers that do not contain inclusions, which is almost reflected in all the reconstruction results. To address this phenomenon, our subsequent work needs to further improve the accuracy of the model in reconstructing the OPs of different layers, including improving the structure of the network and increasing the proportion of experimental data. In future research, our focus will be on enhancing the accuracy of experimental data and devising a sophisticated network architecture to achieve high-quality reconstructions of OPs. Ultimately, the aim is to improve the depth resolution of the reconstructed OPs as well as to optimize the reconstruction accuracy.

## 5 Conclusions

This study employed TR measurements in SFD imaging and effectively applied DL to achieve high-resolution stratified reconstruction of OPs within a 5 mm depth compared with LUT, thereby eliminating aliasing issues associated with $\mu_{a}$ and $\mu_{s}^{\prime }$ during depth reconstruction. Moreover, evaluation metrics further confirmed that DL-based OPs reconstruction showed reduced errors and improved accuracy when using full TR data compared to featured data. In conclusion, the application of DL in TR-SFD imaging enhances resolution and overall accuracy in stratified OPs reconstruction.

## Data Availability

The data that support the findings of this article are not publicly available due to privacy. They can be requested from the author at gaofeng@tju.edu.cn.
